# Transferring Structural Design Principles from Bamboo to Coreless Filament-Wound Lightweight Composite Trusses

**DOI:** 10.3390/biomimetics10120840

**Published:** 2025-12-15

**Authors:** Pascal Mindermann, Martha Elisabeth Grupp

**Affiliations:** 1 Cluster of Excellence Integrative Computational Design and Construction for Architecture (IntCDC), University of Stuttgart, 70174 Stuttgart, Germany; 2 Network for Bio-Inspired Innovations Baden-Württemberg e.V., 68165 Mannheim, Germany; 3 Institute for Textile and Fiber Technologies, University of Stuttgart, 70569 Stuttgart, Germany; 4 Faculty of Technology and Bionics, Rhine-Waal University of Applied Sciences, 47533 Kleve, Germany

**Keywords:** coreless filament winding, bamboo, structural design, fiber composite truss

## Abstract

Bamboo has evolved a highly optimized structural system in its culms, which this study transfers into lightweight fiber composite trusses fabricated by coreless filament winding. Focusing on the structural segmentation involving diaphragms of the biological role model, this design principle was integrated into the additive manufacturing process using a multi-stage winding, a tiling approach, and a water-soluble winding fixture. Through a FE-assisted analytical abstraction procedure, the transition to a carbon fiber material system was considered by determining a geometrical configuration optimized for structural mass, bending deflection, and radial buckling. Samples were fabricated from CFRP and experimentally tested in four-point bending. In mass-specific terms, integrating diaphragms into wound fiber composite samples improved failure load by 36%, ultimate load by 62%, and energy absorption by a factor of 7, at a reduction of only 14% in stiffness. Benchmarking against steel and PVC demonstrated superior mass-specific performance, although mōsō bamboo still outperformed all technical solutions, except in energy absorption.

## 1. Introduction

Fiber composite material systems offer a high degree of adjustability within a multi-parameter design space; enabling the development of highly efficient lightweight structures [1]. Beyond intrinsic material properties, the mechanical performance of these structures is primarily determined by their structural configuration (topology, geometry, and layering). For elongated structural systems subjected to tensile or bending loads, fiber composite profiles with predominantly unidirectional fiber orientation are particularly suitable and have been extensively studied [2,3,4]. Such profiles, characterized by low geometric complexity and unidirectional fiber orientation, can be manufactured cost-effectively by pultrusion [5]. In contrast, more complex profiles are fabricated using filament winding, braiding, or weaving techniques [6]. Due to the bending moment distribution across a profile’s cross-section, material efficiency can be enhanced by employing sandwich design principles [7]. These principles can be easily integrated with the aforementioned manufacturing processes. To improve the mass-specific mechanical performance of such sandwich trusses (or tubes), the outer fiber reinforcement layers can be arranged in a lattice configuration, further reducing material usage [8]. Coreless filament winding (CFW) and hybrid CFW are particularly well-suited manufacturing techniques for producing such lattice truss structures, which have been the subject of extensive research [9,10,11,12,13,14]. However, due to the corresponding reduction in the effective cross-section of the reinforcement material, these structures become increasingly susceptible to other loadings, such as buckling [15].

Bamboo has evolved an efficient structural system in its culms that incorporates diaphragms [16] to prevent ovalization of the tubular structure under load. Adopting a biomimetic top-down approach, the transfer of such structural features into lightweight fiber composite truss structures [14] appears promising as a means of mitigating premature failure with minimal material deployment. Nevertheless, introducing diaphragms similar to those in bamboo into a filament winding process presents significant challenges due to the inherent geometric limitations of current state-of-the-art CFW techniques. Specifically, threading fibers through openings in a fiber net is not feasible within the constraints of the current automated (robotic) CFW [17].

Coreless filament winding [18] is a fiber composite additive manufacturing method that differs from conventional filament winding in that it eliminates the need for a mandrel or core. Instead, it utilizes point-like anchors mounted on an easy-to-adapt fixture [19]. A fiber, (pre-) impregnated with a thermosetting resin, is wound around these anchors in a predefined sequence (winding syntax [20]) and attached using a specific method (hooking syntax [17]), following a three-dimensional winding trajectory [21], usually by an industrial robot [22]. Upon curing the matrix material, the resulting composite structure becomes self-supporting and can be detached from the winding fixture. Winding anchors [23], typically sleeve/washer combinations [24] or metallic pins [25], are affixed to a fixture, usually a reconfigurable metal frame. Recent research [26,27] has minimized even further the fixture by introducing spatial winding and multi-robot systems. In contrast, other studies have sought to increase control by reintroducing guiding surfaces [28,29,30,31] (hybrid CFW) or implementing intermittent curing cycles [13,32] (multi-stage CFW). Despite these advances, the major drawback of CFW remains the significant variation [33] in fabricated components, which originates from insufficient compaction and fluctuations in winding tension [34] as well as in fiber–fiber interaction [35]. As a result, controlling the outer bundle geometry [36], avoiding voids [37], and achieving an evenly distributed fiber volume ratio (FVR) [38] remains challenging. Therefore, a central research objective in CFW is the integration of computational design [39] with process-aware elements, including cyber-physical fabrication [40], digital process characterization [41], integrative design [42], and architectural application [43]. These efforts converge in the development of a co-design methodology grounded in a shared data framework [44], standardized data workflows [45], and fast design feedback [46] in order to improve the FE-assisted [47] predictability of CFW-based lightweight structures in architecture. Accompanying sustainability-focused research enhances the eco-mechanical performance of both fiber [28] and matrix [48] systems. Beyond architectural applications [49,50,51,52,53,54,55], CFW holds promise in high-performance applications [14,31,56], such as aerospace, where material systems such as (pitch-based) carbon fibers are employed for their high stiffness- and strength-to-weight ratios. Moreover, the geometrical configuration of the mechanical system (anchor positioning on the winding fixture) and the digital fabrication parameters (winding and hooking syntax) can be easily adjusted to accommodate the specific loading conditions of each individual component. This flexibility and predisposition for creating spatial structures [57] are the reasons why this study focuses on CFW.

This paper aims to transfer relevant design principles from the structural system of bamboo into a carbon fiber composite lightweight truss realized by coreless filament winding to improve its mass-specific structural performance compared to existing state-of-the-art solutions.

### 1.1. Bamboo/Bambusoideae

Bamboos are a group of fast-growing perennial plants within the grass family (Poaceae), subfamily Bambusoideae [58]. Within the grass family, they are the only group that diversifies in forests and comprise nearly 1500 species [59]. They are classified into two clades—paleotropical woody bamboos and neotropical woody bamboos [58]—with further subdivisions, each with distinct geographic distributions: Bamboo occurs across southeast Asia; central Africa; northeast, central, and South Americas; and northern Australia, with the highest species richness in the Asia-Pacific region; while it is not native to Europe [60]. The plants grow in diverse habitats, from lowlands to elevations exceeding 4000 m, and tolerate environments ranging from humid to arid regions, with extreme temperatures from hot rainforests to approximately −20 °C and annual precipitation up to 1270 mm [60].

Morphologically, bamboo is characterized by hollow, segmented culms [16] extending above ground and underground rhizome systems, which are strongly branched and determine growth patterns [61]. Roots emerge from the rhizome and the bottom of the culm; they generally extend up to 70 cm below the surface [62]. Bamboo exhibits rapid growth, with certain species (*Phyllostachys reticulata* or *Dendrocalamus asper*) growing up to 120 cm in 24 h under optimal conditions [62]. Over longer periods, growth rates (up to 36 m in six months) remain substantially higher than those observed in trees [59]. Compared to other grasses, bamboo exhibits complex branching behavior, with branches usually emerging after the culm reaches its full height [62]. Leaf blades are thin, dorsiventral, and attached by petioles, while many bamboos exhibit leaf dimorphism [61]. Photosynthesis occurs primarily in the leaves and, to a lesser extent, in the culm, which is important during developmental stages [63]. On a chemical level, bamboo consists of long, aligned cellulose fibers embedded in a hemicellulose and lignin matrix [64]. However, compressive strength is approximately twice that of concrete, and ultimate tensile strength is nearly equivalent to mild steel [65].

Ecologically, bamboo serves as a carbon sink [66] and promotes soil stabilization [67]. It contributes to biodiversity by providing habitat for various fauna, including birds and mammals, such as pandas [60]. Economically, bamboo is a vital natural material, with uses similar to timber, and holds cultural, artistic, industrial, and agricultural significance, particularly in rural regions [58]. Its high strength-to-weight ratio and rapid renewability support applications in construction, furniture, flooring [65,67,68], and especially in scaffolding [69]. Bamboo is also used in paper [60], textile [64], and composite [70,71] production. Furthermore, young bamboo shoots are consumed as food and various plant parts are utilized in traditional medicine [72].

### 1.2. Bamboo’s Structural System

**Figure 1:**
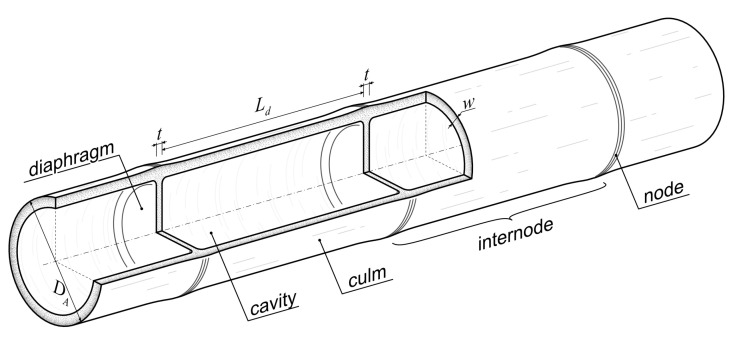
Conceptual drawing of the characteristic bamboo culm’s structural system.

The exceptional structural performance of the bamboo culm arises primarily from its structural configuration (see Figure 1) rather than an inherent superiority of its building materials [73]. Consequently, the bamboo’s structural system is a well-studied [16,73,74,75] model for efficient structural designs. Most relevant load cases of bamboo culm are dynamic lateral cantilever bending under distributed loads, mostly from wind as well as static axial compression by gravity. Bamboo culms reach heights of 5 to 35 m, with an average of 18.3 ± 8.0 m, diameters between 2.5 and 20 cm, averaging 11.6 ± 6.3 cm, and wall thicknesses between 6.7 and 37.5% of the diameter, occasionally solid, with an average of 17.2 ± 8.4%, based on an evaluation of approximately 30 different bamboo species [76]. The culm diameter and culm wall thickness both decrease linearly with height, ensuring that stresses remain constant up to a certain level and subsequently diminish to zero at the tip [16]. The circular cross-section of the culm exhibits a homogeneous bending resistance distribution, thus maximizing axial buckling resistance. As the columns are hollow, they utilize the deployed material most efficiently to counteract bending loads. The outer diameter and wall thickness increase towards the base of the plant to compensate for the increasing bending moments.

Furthermore, the bamboo evolved a structural segmentation of the culm by introducing diaphragms, which transversely connect the inner walls at regular intervals. They can store and release energy from deformations [73]. These additional structural members help the thin-walled tubular culms resist ovalization [74] and thereby delay structural collapse under buckling. The diaphragms function primarily as tension members rather than compression members, as their fibrous material system is more efficiently loaded in tension [77]. The region between two neighboring diaphragms is called the internode; its length varies according to the load distribution along the culm, decreasing toward both the base and the apex [75]. At the apex, new branches require greater support. The axial position of each diaphragm can be identified externally, as a ring-shaped ridge extends beyond the internodial wall diameter at that location on the outer surface of the culm.

At the nodes, the ridge represents a gradual thickening in the culm cross-section, compensating for the local weak point caused by the distortion and redirection of fiber orientations, together with a locally higher second moment of inertia. Fiber orientations deviate at the nodes because the unidirectional fibers in the internodal walls branch partially into the diaphragm; the fiber orientation of the diaphragms does not exhibit a preferred fiber alignment in a transverse cut [78], thereby structurally enabling an effective connection between both types of members at the fiber level. Fibers towards the inside of the culm tend to be 20–40% shorter and in the longitudinal direction, with maximum median length range for one of the internodal lengths [64].

At the fiber-cellular level, bamboo exhibits a functionally graded bauplan [79]: Vascular bundles (VBs) embedded within a parenchymatous tissue matrix, comprising vessels (xylem and phloem) responsible for water and nutrient transport, and enveloped by mechanically supportive sclerenchyma cells. Across the culm cross-section, VBs display a radial gradient characterized by increasing density toward the periphery, accompanied by a decrease in vessel diameter [16]. Consequently, the volume fraction of parenchymatous tissue diminishes accordingly. This graded structural organization ensures that sclerenchyma, the primary load-bearing tissue, is concentrated in regions requiring enhanced mechanical reinforcement, thereby optimizing load absorption efficiency [80]. At the node, VBs implement multiple reinforcement mechanisms [78] to connect the culm and diaphragm, including interlocking, scaffolding, and intertwining architectures. Furthermore, the hollow culm is externally protected by an epidermis and internally by a pith ring, which plays a significant role in the transport of liquids [81].

## 2. Materials and Methods

To validate the transfer of principles and the novel fabrication approach, samples were manufactured and structurally tested. Initially, the relevant features of bamboo were analyzed, selected, and abstracted for transfer. Subsequently, the geometrical parameters optimizing bending stiffness were identified, considering the transition from bamboo’s biological building materials to carbon fiber-reinforced plastic (CFRP). Next, the winding syntax was defined based on processing characteristics and sample dimensions constraints consistent with testing capabilities. Finally, the samples underwent destructive testing. For comparison, a CFW sample without bamboo-inspired features, a steel square tube, a PVC pipe, and a mōsō bamboo (*Phyllostachys edulis*) culm was also experimentally tested.

### 2.1. Feature Selection

The technical adaptation of bamboo’s structural principles incorporates several key features while excluding others due to limitations in sample length, process complexity constraints, or lack of structural relevance. Plants and animals must fulfill multiple functions, such as water and nutrient transport and self-healing processes [82], rather than maximizing a single function [83]. Consequently, the complete transfer of all bamboo features into a technical adaptation is neither feasible nor desirable, as some features serve primarily biological roles. Therefore, only the most structurally relevant and transferable features for CFW were selected for abstraction; see Table 1.

For the realization in the case study, the adapted features include an approximation of the circular, hollow cross-section and an almost uniform wall thickness. Diaphragms are incorporated into the design, and the outer wall thickening at the node is achieved by tangentially reinforcing the node areas with additional circumferential winding layers. The system preserves uninterrupted fiber paths between diaphragms and internodes, incorporates unidirectional fiber layers in the internodes, and integrates fibers oriented at various angles within the diaphragms. The use of fiber-reinforced composite material reflects the fibrous arrangement found in natural bamboo. A radial gradient in fiber distribution is approximated through the defined winding syntax.

In contrast, axial variations in diameter and wall thickness, as well as internodal length variations, are not incorporated because the sample is too short. Several biological features are not adapted due to implementation complexity, including diverse reinforcement schemes within VBs, the intentional bundling of vessels and sclerenchyma cells, and gradients in vascular bundle diameter. Furthermore, the epidermal layer and the pith ring are omitted owing to their limited relevance to the current structural application.

### 2.2. Feature Abstraction

As the biological model and the technical adaptation do not utilize the same material system (see Section 2.3) it is necessary not only to replicate the geometrical blueprint and its length relationships but also to adapt the principle to the new material system by identifying the optimal dimensional configuration. For the case study, the nominal outer diameter of the samples was arbitrarily set to 100 mm. Based on this, optimal values for the culm wall thicknesses and diaphragm geometry were determined through numerical simulations and analytical calculations exploring the feasible design space. The investigated ranges of the design space were selected based on values found in bamboo. Since failure behavior in CFW structures is difficult to model with sufficient predictive accuracy, especially given the complex fiber orientations that cause significant discrepancies between models and actual objects post-manufacturing, an analysis of strength and failure was not conducted. Instead, evaluations were limited to linear elastic calculations. These FEAs only assess the linear elastic deformation of the model for a given parameter set while considering the mass of the structure to achieve a lightweight design. First, the influence of wall thickness *w* was investigated by simulating a cylinder without diaphragms. In a second run, the number *n* and thickness *t* of diaphragms were varied for the found wall thickness.

#### 2.2.1. FE Model and Design Space Ranges

To reduce computational cost, the FE model was simplified and consists of a hollow cylinder with an outer diameter $D_{A}$ of 100 mm and a fixed total sample length $L_{D}$ of 1500 mm. Wall thickness *w* and the number *n* and thickness *t* of diaphragms were varied discretely within individual ranges: The investigated wall thickness ranges from 5 mm to 45 mm, slightly extending the range in both directions observed in bamboo species of equivalent diameters [76]. Based on internode lengths reported for various bamboo species, the number of diaphragms (only uneven numbers) ranged from 3 to 15 at equal distributions along the total length. Results were then interpolated to include even *n* numbers and later extrapolated to a range of 1 to 20 diaphragms. For *t*, a broad range from 1 to 10 mm was selected. As the FEA simplifies the complex bamboo structure, diaphragm thicknesses and internode lengths were held constant along $L_{D}$. The boundary conditions consisted of fixed supports at both ends of the culm, and a uniform load $F_{D}$ of 20 kN was distributed across the entire length $L_{D}$. The average FE mesh size was approximately 20 mm.

#### 2.2.2. Material Properties

The material properties assigned to the FE model’s outer cylinder, representing the culm, are anisotropic standard CFRP parameters accounting for the unidirectional fiber orientation. For the isotropic diaphragms, the anisotropic material parameters in the fiber direction were reduced to approximate the random fiber orientation observed in the diaphragm of the biological reference model. The modulus of elasticity *E* based on the angular deviation θ between the load vector and fiber orientation [84] can be calculated based on axial and shear moduli of elasticity and Poisson’s coefficient. Expanding on this and assuming an equal distribution of fiber orientation angles, the reduced modulus of elasticity $\hat{E}$ for the diaphragm can be calculated by

(1) $$\hat{E}=\frac{2}{\pi }\int_{0}^{\frac{\pi }{2}}\frac{1}{\frac{1}{E_{x}}c^{4}+\left(\frac{1}{G_{xy}}-2\frac{\nu_{xy}}{E_{x}}\right)s^{2}c^{2}+\frac{1}{E_{yz}}s^{4}}\;d\theta$$

with trigonometric functions sin(θ) and cos(θ) denoted with *s* and *c* and with

$E_{x}$ axial modulus of elasticity in fiber direction [GPa], here 121 GPa;
$E_{yz}$ axial modulus of elasticity transverse to fiber direction [GPa], here 8.6 GPa;
$G_{xy}$ shear modulus of elasticity [GPa]; here 4.7 GPa;
$\nu_{xy}$ Poisson’s coefficient [-], here 0.27;

resulting in a $\hat{E}$ of 26.8 GPa.

#### 2.2.3. Determination of Wall Thickness

**Figure 2:**
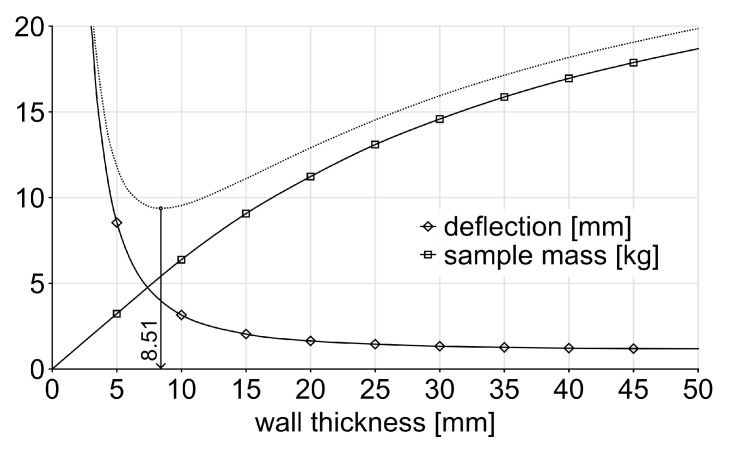
FEA results showing the impact of wall thickness on bending deflection and sample mass. Sample length equals 1500 mm. Optimum results at 8.51 mm is determined from the minimum of the unweighted sum of deflection and mass.

For the determination of the optimal wall thickness of the culm, the diaphragms are excluded. The mass of structure as well as the maximum deflection in y direction was numerically calculated for the given range in relevant wall thicknesses; see Figure 2.

The results were approximated by a symmetrical sigmoidal function following this general form:

(2) $$f\left(x\right)=d+\frac{a-d}{1+{\left(\frac{x}{c}\right)}^{b}}$$

with *x* as the wall thickness of the culm *w*. For the deflection of the culm without diaphragms, the following parameters were found: a = 12,793,590 ± 4.658 × 10^12^; b = 1.841681 ± 0.2381; c = 0.002077834 ± 410.8; d = 1.051195 ± 0.06689; R^2^ = 99.98%. For the mass of the culm without diaphragms, the following parameters were found: a = 0.03174107 ± 0.1202; b = 1.189712 ± 0.05256; c = 28.20321 ± 2.793; d = 28.18243 ± 1.633; R^2^ = 99.97%.

With increasing wall thickness, the deflection decreases, but the mass of the structure also increases. Above a certain value, only a small further reduction in deflection for big increases in mass is possible. Both deflection and mass should be equally minimized. There are several options in multi-objective optimization for finding the optimum wall thickness based on deflection and mass, such as finding the intersection or minimizing the product. Here, the optimum wall thickness was found at *w* = 8.51 mm as the minimum of the unweighted sum of deflection and mass.

#### 2.2.4. Determination of Diaphragm Parameters

To determine the optimum combination of number and thickness of diaphragms for the carbon fiber material system, several application-relevant influences were analytically or numerically analyzed at a constant wall thickness of the culm, as established in the previous step:

M(n,t) mass of the structure (analytical);
D(n,t) maximum bending deflection under line load (numerical);
$F_{d/b}\left(n,t\right)$ ratio between diaphragm’s partial load and max. buckling force (analytical);
Q(n,t) eigenfrequency of the structure (numerical);

with diaphragm thickness *t* ranging from 1 to 10 mm and the number of diaphragms *n* ranging from 1 to 20.

##### Structural Mass

The structural mass of the system was calculated from the volume and the average density of the CFRP material according to the following equation:

(3) $$M\left(n,t\right)=M_{0}+M_{d}\;n\;t$$

with

*M* 
total structural mass [kg];
$M_{0}$ 
mass of the culm [kg] without diaphragms, here 5.487 kg;
$M_{d}$ 
mass of an individual diaphragm of 1 mm thickness [kg], here 4.975 g;
*n* 
number of diaphragms [-];
*t* 
(average) diaphragm thickness [mm].

Within the investigated ranges, the diaphragms provide only a minor contribution to the total structural mass.

##### Bending Deflection

**Figure 3:**
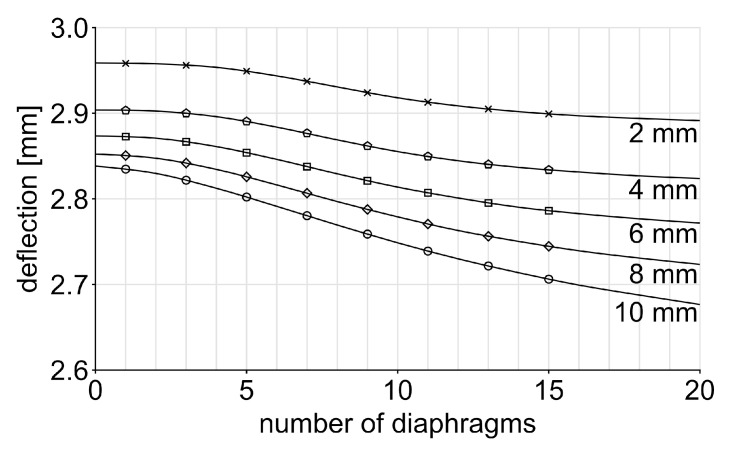
FEA results showing the impact of diaphragm number and thickness on bending deflection. Thicknesses below 2 mm result in deflections above 3 mm.

The bending deflection D(n,t) was determined by FE analysis (see Section 2.2.1) for the ranges of diaphragm numbers and thicknesses (see Figure 3). As the number *n* and thickness *t* of diaphragms increase, the deflection *D* decreases, while the structural mass increases with *n* and *t*, compare with Equation (3). The deflection follows a nonlinear curve, exhibiting a steeper decline within the middle of the investigated range for *n*, whereas changes in *t* produce only gradual variations. Increasing diaphragm thickness yields diminishing returns in reducing deflection at the upper end of the investigated range. The impact of diaphragm thickness on deflection also becomes more pronounced as the number of diaphragms rises.

The simulation result (see Figure 3), was approximated using again a symmetrical sigmoidal function (see Equation (2)), through a two-step procedure: First, the deflection $D_{t}\left(n\right)$ was approximated as a function of the number of diaphragms, producing one equation per diaphragm thickness. Second, the coefficients *a* through *d* of these equations were subsequently approximated as functions of diaphragm thickness by applying Equation (2) again, resulting in an approximation of D(n,t); see coefficients in Table 2.

##### Radial Buckling

In addition to bending, buckling is a critical failure mechanism, particularly for thin-walled and filigree structures. For applications [14] where axial compression loads are of secondary importance, culm buckling is negligible; therefore, only radial buckling, corresponding to diaphragm buckling, was considered in the design. In the natural role model, the isotropic material properties (in the y,z plane) and the higher load-bearing capacity of the fibrous material in tension than in compression result in diaphragms being primarily tension-loaded, rendering buckling of minor relevance [77]. However, the technical adaptation does not allow for the same level of homogeneousness in fiber distribution in the diaphragms; thus, buckling may occur in the technical adaptation and is evaluated.

For the calculation, an Euler buckling model with both ends fixed in rotation and translation was assumed for each diaphragm, analyzing the structure from the perspective of a radial cut. Deviating from classical Euler assumptions, the second moment of inertia was computed by averaging the variable diaphragm width over its height, with *h* calculated as

(4) $$h=\frac{\pi }{16}\;{\left(D_{A}-2w\right)}^{3}$$

with

$D_{A}$ 
culm outer diameter [mm];
*w* 
culm wall thickness [mm].

A rectangular cross-section was assumed for the second moment of inertia. The Euler model assumes homogeneous and isotropic material, which the technical diaphragm does not fully satisfy. For the calculated critical buckling load, the diaphragm E-modulus $\hat{E}$ was used, and the resulting load was compared to the corresponding partial axial load $F_{d}$ with

(5) $$F_{d}=F_{D}/n.$$

Combinations of diaphragm number *n* and thickness *t* for which the critical load $F_{b}$ was smaller than the partial load $F_{d}$ were excluded from the solution set. This situation occurred for 1 mm thick diaphragms with fewer than n=3.

(6) $$F_{d/b}\left(n,t\right)=\frac{F_{d}\left(n\right)}{F_{b}\left(t\right)}=\frac{3}{4\;\pi^{2}}\frac{F_{D}\;{\left(D_{A}-2w\right)}^{2}}{E\;n\;h\;t^{3}}$$

with

$F_{d}$

axial load per diaphragm [N];

$F_{b}$

Euler’s buckling load (rotation and translation fixed at both ends) [N];

$F_{D}$

accumulated line load along culm [N], here 20 kN;

$D_{A}$

outer diameter of the culm [mm], here 100 mm;
*w* 
wall thickness of the culm [mm], here 8.51 mm, see Section 2.2.3;
*E* 
elastic modulus of the diaphragm’s material [N/m^2^], see Equation (1);
*n* 
number of diaphragms [-];
*h* 
width of the diaphragms [mm], see Equation (4);
*t* 
thickness of the diaphragms [mm].

##### Eigenfrequency

The eigenfrequencies were determined by FE analysis (see Section 2.2.1) for different combinations of the diaphragm parameters *n* and *t*. No significant changes or extractable trends were observed within the investigated range for either parameter. Consequently, only a subset of the full range was explored: *t* values of 2 and 8, as well as *n* values from 1 to 9, were simulated. Averaging the first eigenfrequency over this reduced ranges yields

(7) Q(n,t)=469.05±5.04Hz.

Therefore, the eigenfrequency is not further considered for the selection of *n* or *t*. The eigenmode associated with the first eigenfrequency resembles that of global three-point bending, and the obtained eigenfrequency values indicate adequate structural performance.

##### Weighting of Influences

At the optimal number of diaphragms *n* for each *t*, the weighted sum of M(n,t), D(n,t), and $F_{d/b}\left(n,t\right)$ becomes minimal. Over the investigated design space for *n* and *t*, M(n,t) ranged from 5.492 to 6.482 kg, D(n,t) from 3.427 to 2.675 mm, and $F_{d/b}\left(n,t\right)$ from 382.3% to 0.02%. Since the absolute variation in buckling was several orders of magnitude larger than that of mass or deflection, a direct comparison would have been biased toward buckling. To account for this, all variables were first normalized over their functional value ranges, so that only the relative trends, rather than absolute magnitudes, contributed to the weighted sum optimization approach. Thus, the functional values for each of M(n,t), D(n,t), and $F_{d/b}\left(n,t\right)$ were normalized over the investigated design space ranges *n* = 1 to 20 and *t* = 1 to 10, by applying

(8) $$\hat{x}=\frac{x-\min(x_{n,t})}{\max\left(x_{n,t}\right)-\min\left(x_{n,t}\right)}$$

with *x* as the respective functional values of M(n,t), D(n,t), and $F_{d/b}\left(n,t\right)$.

For deflection and buckling, a weighting factor of 31.7% was applied, while a slightly larger weighting factor of 36.6% was used for mass. While an equal weighting scheme (33.3% for each) could have been applied, the chosen weighting factors slightly favor mass. However, the difference between the weighting factors is deliberately kept minimal with only one additional 1/10 for mass (mass +3.3%) so that it does not dominate stiffness/functionality (deflection) and stability/safety (buckling) in the optimization.

While the investigated design space is based on the natural role model, for the subsequent CFW fabrication process, only diaphragm thicknesses at the lower end of the investigated range appear feasible to achieve. Following the same approach as in Section 2.2.3, the minimum of the weighted sum was searched for selecting *n* and *t*. With this weighting, *n* = 15 diaphragms are ideal for *t* = 1 mm thickness, *n* = 6 diaphragms for *t* = 2 mm thickness, and *n* = 2 diaphragms for *t* = 3 mm thickness. As diaphragm thickness increases, the required number of diaphragms decreases drastically, indicating that a larger number of thinner diaphragms is structurally more efficient.

##### Generalization

A diaphragm thickness *t* of approximately 2 mm was targeted for the later fabrication of the samples. Since the required number of diaphragms depends on the total length, the selected optimum must be converted into the corresponding internode length. The internode length (measured between diaphragm surfaces, not between midpoints) is given by

(9) $$L_{d}=\frac{L_{D}-n\;t}{n+1}$$

with

$L_{d}$ 
internode length, accounting for diaphragm thickness;
$L_{D}$ 
total culm length;
*n* 
number of diaphragms (no diaphragms at culm end);
*t* 
diaphragm thickness.

For $L_{D}$ = 1500 mm, *t* = 2 mm, and *n* = 6, the resulting internode length is $L_{d}$ = 212.6 mm. For sample fabrication, the following geometrical parameters were targeted relative to the outer culm diameter ($D_{A}$ = 100%): *w* = 8.5% wall thickness; *t* = 2% diaphragm thickness; $L_{d}$ = 212.6% internode length, (compare Figure 1).

### 2.3. Material System

#### 2.3.1. Fiber System

Coreless filament winding can process both natural and synthetic fibers. Carbon fibers were selected due to their excellent mass-specific mechanical performance and the envisioned lightweight applications. A 12K HTS carbon fiber with a linear density of 800 tex, tensile modulus of 240 GPa, tensile strength of 4400 MPa, and density of 1.77 g/cm^3^ was chosen. The elastic modulus and strength of bamboo fibers are 46 GPa and 600 MPa, respectively [85]; thus, in comparison, carbon fibers are 5.2× and 7.3× more performative, respectively, than the bamboo fiber. Lower linear density values typically correspond to a more homogeneous fiber layup, but they also increase fabrication time linearly. Pitch-based carbon fibers offering even higher mechanical performance and increased processing challenges [14] were not selected due to cost-efficiency considerations: Material selection is of secondary importance in this case study, which focuses on the development of the manufacturing concept for integrating a bamboo-inspired building system into CFW, where materials can be easily substituted later without altering the process or equipment.

For additional reinforcement at load distribution points, a unidirectional carbon fiber tape was utilized as surface reinforcement due to its excellent drapability. This pitch-based carbon fiber tape features fibers aligned unidirectionally on a polyester fiber net, with a tensile modulus of 760 GPa and a tensile strength of 3200 MPa. The tape was selected based on availability and its prior effective use with CFW, as documented [14].

#### 2.3.2. Matrix System

The CFW process requires thermoset matrices; for this study, an epoxy resin widely used in CFW was employed. It exhibits high mechanical properties, effective fiber impregnation, a viscosity within the ideal processing range, and a sufficiently long pot life. The selected two-component resin has a viscosity of 430 mPas at a density of 1.18–1.20 g/cm^3^, achieves a flexural modulus of 2.7–3.2 GPa, a flexural strength of 90–120 MPa, and a pot life of approximately 300 min. The bamboo matrix tissue [85] reaches values around 2 GPa and 50 MPa for modulus and strength, respectively; thus, epoxy resin is 1.5× and 2.3× times more performative than the bamboo matrix. Comparing fiber and matrix, the ratio in mechanical performance between the fiber and matrix is much larger in the technical adaptation than in the natural role model.

### 2.4. Sample Fabrication

#### 2.4.1. Sample Plan

**Figure 4:**
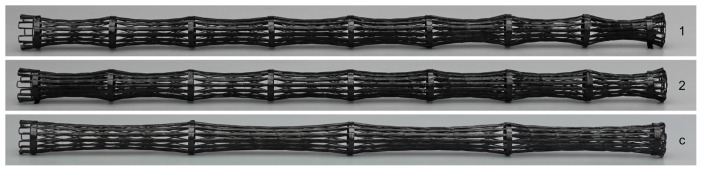
Overview photos of bamboo-inspired (samples 1 and 2) and comparison (c) CFW samples.

Due to the extensive manual labor required for manufacturing the bamboo-inspired CFW samples and multiple iterations needed to produce fully functional samples, after the design freeze, only two samples were identically fabricated to demonstrate the feasibility of the concept; see samples 1 and 2 in Figure 4. For comparison, a CFW sample without diaphragms was also fabricated and tested; see (c) in Figure 4.

#### 2.4.2. Tiling into Subcomponents

The integration of diaphragms into the CFW structure requires novel modifications to the conventional CFW process. Typically, the rovings already suspended between anchors obstruct the placement of fibers going beneath them, making it impossible to wind diaphragms without threading the fiber through openings, which is an operation that state-of-the-art winding equipment does not allow. This study introduces a novel extension to the multi-stage CFW process by combining several stages with a tiling approach. This method enables the fabrication of diaphragms with improved structural integration through strategic seam placement, surpassing conventional differential construction methods where diaphragms are attached later to a cylinder without continuous fiber bridging between the two structural entities (e.g., continuous rovings connecting the culm and diaphragms). Subcomponents fabricated during early stages are subsequently joined in later stages of the multi-stage CFW winding process. Each subcomponent comprises a segment of the diaphragm and a portion of the cylindrical hull. These subcomponents have a diaphragm at both ends, each with half the diaphragm thickness. This configuration was selected as it seems to be superior to an alternative design featuring a single full diaphragm and a portion of the culm, which might be less geometrically stable during reconfiguration and more challenging to design with a homogeneous material distribution. In addition, positioning the seam within the diaphragm increases the available bonding surface.

#### 2.4.3. Winding Frame

A central aspect in realizing diaphragms within CFW truss structures is the design of the winding frame, particularly its removability after winding and its capacity to enable the joining of subcomponents. In state-of-the-art processes, diaphragms would geometrically obstruct access to the winding frame after placement, preventing its removal by disassembly or destruction. Moreover, conventional winding frames rigidly fix the winding anchors in space throughout the entire fabrication session, disallowing the reconfiguration required for subsequent winding stages necessary to join subcomponents.

**Figure 5:**
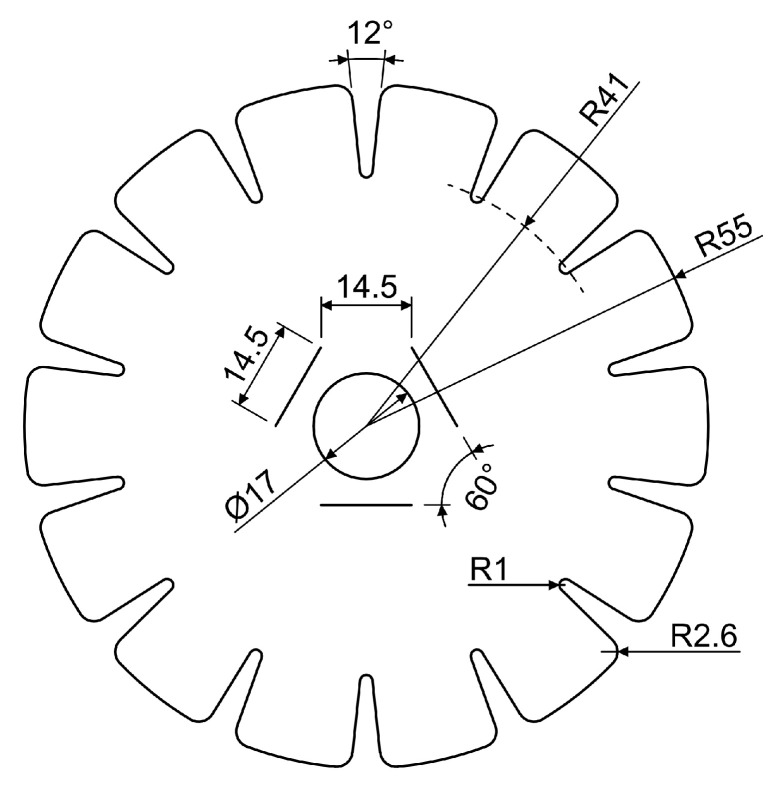
Cardboard disk used for fiber anchoring. Dimensions in mm.

In this study, the proposed solution combines a water-soluble holder with the capability to move these holders along one dimension, enabling the required reconfiguration. Several materials can serve as water-soluble holders with adequate mechanical performance to withstand the winding process and no adverse interactions with epoxy resin. Cardboard was selected in this study due to its suitability for laser cutting in large quantities with high geometric precision. Both (3D-printed) PVA and cardboard can be dissolved in water; however, cardboard offers greater cost-efficiency, and the cut-outs can be rapidly produced using a cutting plotter (see Figure 5). The cardboard used has a thickness of 2 mm. Beeswax was selected as a release agent for the cardboard based on initial testing.

**Figure 6:**
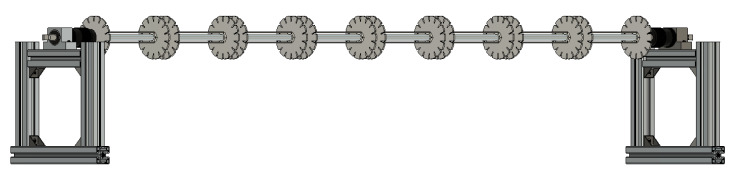
Winding fixture for the bamboo-inspired CFW samples. For the fabrication of the comparison CFW sample, only five equally distanced cardboard disks were lined up.

A 2 m long threaded rod with a diameter of 16 mm served as the backbone of the winding frame and was secured at both ends in chucks to allow rotation during the winding process; see Figure 6. A circular cut-out was incorporated at the center of the cardboard disks to accommodate the anchors on the threaded rod. Since the 2 mm thick cardboard alone cannot withstand the fiber tension during winding, two layers of cardboard were glued together for reinforcement. Water-soluble wood glue was employed to facilitate the removal of the cardboard after curing. To spatially fix the anchors, hexagon nuts were attached to the cardboard using epoxy glue. Additional cuts were introduced into every second cardboard piece to improve the positioning reliability of the hexagon nuts. Two disks are required to wind one subcomponent, positioned according to the internode distance. To enable sliding of the subcomponents along the threaded rod for connecting all uncured subcomponents after the first winding stage, relative movement between anchors within each subcomponent must be prevented to avoid twisting of uncured fiber bundles. This constraint is enforced by joining the hexagon nuts together using small cardboard rectangles bonded with epoxy glue; see Figure 6. For the rod, a state-of-the-art mold release agent was used.

#### 2.4.4. Winding Anchors

**Figure 7:**
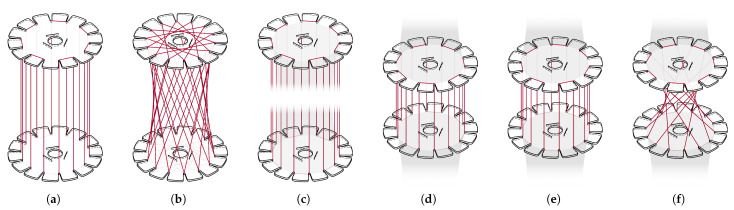
Winding subsyntaxes for the bamboo-inspired CFW samples; compare with Table 3. (**a**) Subsyntax 1.1. (**b**) subsyntax 1.2. (**c**) subsyntax 2.1. (**d**) subsyntax 2.2. (**e**) subsyntax 2.3. (**f**) subsyntax 2.4. Anchors are numbered consecutively, from 1 to 14 on one side of the internode and from 15 to 28 on the other one in the same rotational direction, with node 1 and 15 opposite each other. In the comparison CFW sample, this numbering continues across all disks, with the highest anchor number at node 70. The distance between adjacent disks in stage two is not shown to scale for better visibility.

Winding pins were not used; instead, a different type of winding anchor is employed: a slit designed to hold the fibers within the cardboard disks. This approach reduces volume and can be directly integrated via CNC cutting. The number of winding anchors critically determines the outer geometry of the structure. A cylindrical shape cannot be realized with too few winding anchors, whereas an excessive number of anchors reduces the spacing between them, thereby limiting the available area for placing rovings. The possible fiber angles are directly influenced by the number of anchors. While the number of anchors cannot be increased arbitrarily, an increase in the number of anchors translates into a more effective approximation of the quasi-isotropic character of the diaphragm. Furthermore, especially within the main syntax, an improved force distribution and reduction of local buckling can be achieved by increasing the number of fiber crossings, defined as the intersections of two rovings. Several configurations with varying anchor counts were developed to identify an optimal number that enables winding a quasi-isotropic diaphragm with low fiber angles and numerous crossings. Fourteen anchors per disk proved to be an effective compromise as a tangential shift of three between both disks provides sufficient crossings to support the UD fiber layers of stage two; see Figure 7.

#### 2.4.5. Winding Syntax

Since a small gap always exists between adjacent winding anchors, the lateral surface of the component is not entirely continuous, negatively affecting force distribution in the tangential direction. Furthermore, a purely unidirectional fiber arrangement increases the risk of local buckling of individual separated fiber bundles. Bamboo exhibits anisotropic fiber orientation along the culm, resulting in maximum stiffness and strength in the longitudinal direction. Therefore, the majority of fiber bundles in the technical composite should be aligned and tensioned between anchors along the longitudinal axis. However, to improve force distribution between rovings and reduce the risk of local buckling of potentially separated fiber bundles, additional fiber angles should be introduced. These deviations from 0° must be strictly limited, as any departure from the longitudinal fiber direction directly decreases strength and stiffness in axial loading. Improving force distribution is especially important under compression. At a tangential shift of three, the quasi-isotropic diaphragm can be wound transversely with a single continuous fiber, if the rotational winding direction inverts at the halfway point of the second subsyntax of stage 1. As the winding anchors are slits, there is no hooking syntax to discuss.

**Table 3:** Winding syntax of the CFW samples. Asterisks indicate anchors in the 8th subcomponent, and underlined indices denote anchors in the neighboring subcomponent.

Sample Type	Stage	Subsyn.	Subcomp.	Repet.	Winding Sequence
bamboo-inspired sample	1	1.1	each indiv. subcomp. 1 to 8	2	1, 15, 16, 2, 3, 17, 18, 4, 5, 19, 20, 6, 7, 21, 22,
					8, 9, 23, 24, 10, 11, 25, 26, 12, 13, 27, 28, 14, 1
		1.2	each indiv. subcomp. 1 to 8	2	1, 18, 23, 12, 3, 20, 25, 14, 5, 22, 27, 2, 7, 24,
					15, 4, 9, 26, 17, 6, 11, 28, 19, 8, 13, 16, 21, 10,
					1, 26, 21, 4, 13, 24, 19, 2, 11, 22, 17, 14, 9, 20,
					15, 12, 7, 18, 27, 10, 5, 16, 25, 8, 3, 28, 23, 6, 1
	2	2.1	across all subcomp., hooking at 1 and 8	18	1, 15 *, 16 *, 2, 3, 17 *, 18 *, 4, 5, 19 *, 20 *, 6, 7,
					21 *, 22 *, 8, 9, 23 *, 24 *, 10, 11, 25 *, 26 *, 12,
					13, 27 *, 28 *, 14, 1
		2.2	between subcomp.	3	15, 1, 14, 28, 27, 13, 12, 26, 25, 11, 10, 24, 23,
			(sin-cos)		9, 8, 22, 21, 7, 6, 20, 19, 5, 4, 18, 17, 3, 2, 16, 15
		2.3	between subcomp.	3	28, 14, 13, 27, 26, 12, 11, 25, 24, 10, 9, 23, 22,
			(offset sin-cos)		8, 7, 21, 20, 6, 5, 19, 18, 4, 3, 17, 16, 2, 1, 15, 28
		2.4	between subcomp.	2	15, 10, 9, 18, 17, 12, 11, 20, 19, 14, 13, 22, 21,
			(circumferential)		2, 1, 24, 23, 4, 3, 26, 25, 6, 5, 28, 27, 8, 7, 16, 15
comparison sample	1	1.1	-	2	1, 57, 58, 2, 3, 59, 60, 4, 5, 61, 62, 6, 7, 63, 64,
					8, 9, 65, 66, 10, 11, 67, 68, 12, 13, 69, 70, 14, 1
		1.2	-	2	1, 21, 41, 47, 67, 58, 50, 42, 20, 12, 3, 23, 29, 49,
					69, 60, 52, 30, 22, 14, 5, 25, 31, 51, 57, 62, 54, 32,
					24, 2, 7, 27, 33, 53, 59, 64, 56, 34, 26, 4, 9, 15, 35,
					55, 61, 66, 44, 36, 28, 6, 11, 17, 37, 43, 63, 68, 46,
					38, 16, 8, 13, 19, 39, 45, 65, 70, 48, 40, 18, 10, 1,
					23, 31, 53, 61, 70, 50, 30, 24, 4, 13, 21, 29, 51, 59,
					68, 48, 42, 22, 2, 11, 19, 41, 49, 57, 66, 46, 40, 20,
					14, 9, 17, 39, 47, 69, 64, 44, 38, 18, 12, 7, 15, 37,
					45, 67, 62, 56, 36, 16, 10, 5, 27, 35, 43, 65, 60, 54,
					34, 28, 8, 3, 25, 33, 55, 63, 58, 52, 32, 26, 6, 1
	2	2.1	-	18	1, 57, 58, 2, 3, 59, 60, 4, 5, 61, 62, 6, 7, 63, 64,
					8, 9, 65, 66, 10, 11, 67, 68, 12, 13, 69, 70, 14, 1

#### 2.4.6. Fabrication Stages

**Figure 8:**
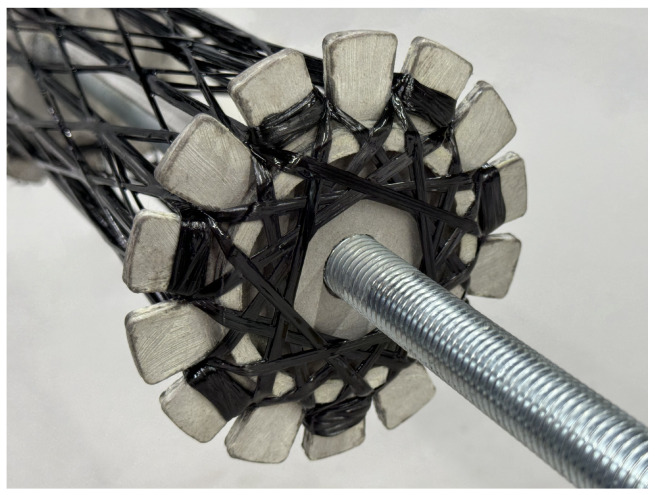
Half of a wound diaphragm completed after the first stage, hold in place by the cardboard disk. The added cardboard ring was removed in later iterations.

After applying the release agents, all anchor disks are arranged on the threaded rod via their nuts and connected by the cardboard rectangles; see Figure 6. To facilitate diaphragm winding, spacing is maintained between adjacent anchor disks. The roving is impregnated and manually placed according to the winding syntax; see Table 3. Upon completion of this first winding stage; see Figure 8, the individual subcomponents are rotated around the threaded rod to bring their diaphragm surfaces into contact. Next, to prevent deformation of the rovings placed in the first stage during the second stage, a partial curing period of 12 h at room temperature is implemented between stages.

Subsequently, the UD fiber bundles are tensioned over the entire length of the structure during the second winding stage. Given that each subcomponent exhibits a tapering geometry due to the shift angle resulting from subsyntax 1.2, the rovings tensioned in the second stage do not contact the cured rovings from the first stage. To improve force distribution throughout the fiber nets of both stages by establishing mechanical contact, a sewing yarn is helically wrapped around the entire structure with an average slope of 35 mm. Since the rovings from stage one are already cured, only the freshly placed rovings from stage two undergo deformation during this wrapping and contact is established.

**Figure 9:**
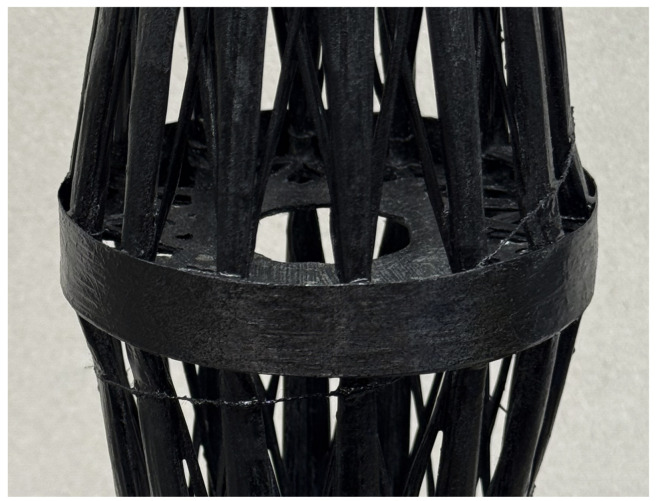
Bamboo-inspired diaphragm made by CFW and reinforced by CFRP tape.

Due to the ratio between the total sample length and the additional roving length required in stage two to accommodate this deformation, fiber tensions would increase to inappropriate levels if sufficient excess material were not provided. This additional length, approximately 26 mm, was determined during earlier iterations of the manufacturing process development. To supply this extra length, the rovings are extended beyond the actual structure length and anchored at an additional anchor disk placed beyond on each end of the sample. During the helical wrapping following stage two, the stage two rovings are released from the anchors on one side to provide slack for the pressing action, while on the opposite side, the rovings remain anchored. By pulling the disk with the anchored fiber bundles, tension is applied to the fiber bundles, ensuring proper alignment and consolidation. As an optional step, after the helical wrapping is completed, potential excess fiber material can be pressed onto the outside of the outer diaphragm to enhance the cosmetic appearance. As a last processing step before curing, a pitch-based carbon fiber tape (see Section 2.3) was draped circumferentially around the nodes and impregnated with the epoxy resin using a brush to allow proper load induction during the four-point bending testing and effective tangential load distribution in the node region (see Figure 9).

### 2.5. Testing Setup and Parameters

**Figure 10:**
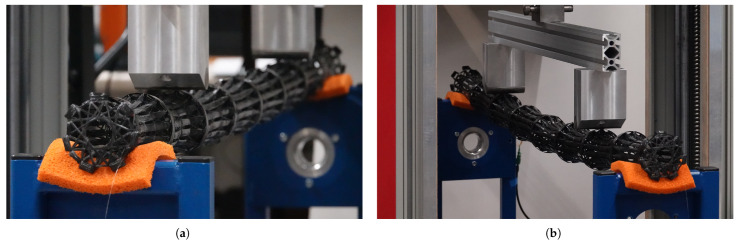
Test setup. (**a**) Before and (**b**) during the testing of the bamboo-inspired CFW samples (sample number 2).

Custom destructive structural testing was performed. To ensure a constant bending moment, a four-point bending setup was realized using a 100 kN spindle-driven universal testing machine combined with custom supports, necessitated by the sample lengths exceeding the equipment’s supported dimensions (see Figure 10). The samples were supported from below at the center of the outermost internodes, as placing a support directly at the outermost diaphragm position risked sample slippage during testing. To further prevent slippage, 20 mm thick foam rubber pads were placed between these supports and the internodes. The load was applied from above by the universal testing machine at the diaphragm positions corresponding to the 2–3 and 6–7 internodes. The spacing between the inner supports encompassed four internodes, while the distance between each inner support and the respective outer support included two internodes. The distance between the supports was individually adjusted for each sample to compensate for fabrication variations, but remained constant throughout each test. The support diameter was 30 mm.

**Figure 11:**
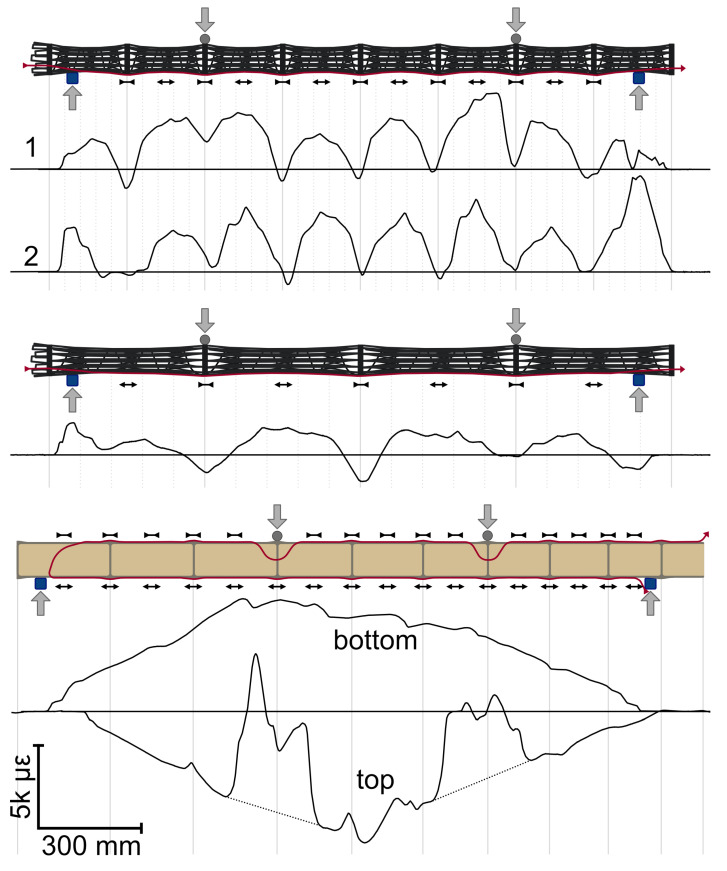
Strain along the bottom (and top) of the samples immediately before failure. Path of the sensor marked in red. Black arrows indicate sign of the local strain.

Testing was conducted under position control at a loading rate of 4 mm/min and was terminated upon significant structural failure. Force and displacement data were recorded continuously. To measure strain along the sample during testing, a fiberoptic sensor interrogated by a Rayleigh-based system was employed [86]. A sensor with an acrylate coating was used. The sensor was glued to the outer surface of the sample using fast-curing epoxy, following a stage two fiber bundle along the sample. When approaching the ends of the sample, the sensor was placed more laterally to avoid interfering with the supports. The sample was oriented so that the sensor was located at the bottom during testing. For the non-CFW samples, no fiberoptic sensor was used, except for the mōsō bamboo culm, where it was also attached to the outer surface by gluing, but along a different path; compare Figure 11. The preload was set to 100 N for CFW samples, 30 N for the mōsō bamboo, and 20 N for all other samples.

### 2.6. Sample Parameters

During fabrication (see Table 4) fiber consumption was determined by measuring the difference in spool weight before and after winding, accounting for cut-offs. Using this value along with the sample weight, the matrix mass was calculated. Subsequently, the FVR was computed through density conversion equations. The FVR was evaluated for each winding stage, revealing a lower FVR in the initial stages of the bamboo-inspired CFW samples. This reduction results from decreased winding speed caused by more complex winding trajectories during the first stage. Conversely, the comparison sample exhibited a higher FVR, which aligns with its simpler winding process relative to the bamboo-inspired specimens. A reduced FVR might suggest an increased void ratio.

Furthermore, the geometric dimensions of the samples were measured; see Table 5. The nominal dimensions of the CFW samples include an outer diameter of 100 mm, an internodal length of 209.8 mm, and a total length of 1720 mm. The deviations between nominal and measured dimensions fall within the expected range for CFW and are attributed to the rigidity of the winding frame relative to the applied winding tension.

## 3. Results and Discussion

In addition to the CFW samples, a steel square tube and a PVC pipe were tested under identical support conditions. Also, a mōsō bamboo culm was tested for comparisons to the biological role model with support conditions as similar as possible; compare Figure 11. The mōsō bamboo culm was inspected to ensure the absence of visible outer cracks, which was to prevent such defects from such defects from potentially influencing the test results. Mass and dimensions were also measured for these samples; see Table 5. The strain distribution along the samples, failure behaviors, and absolute as well as mass-specific mechanical performance were analyzed.

### 3.1. Strain Measurement

The strain along the placed fiberoptic sensor (see Section 2.5) was recorded during testing until the sensor failed due to rupture. The strain values immediately before failure were mapped to the geometry; see Figure 11. The mapping is suitable for comparisons, but this visualization does not account for deviations in the internodial lengths of the CFW samples (see Table 5) the lateral placement of sensors to avoid the lower supports (see Figure 10) and the additional sensor length introduced by detours along the mōsō bamboo sample to bypass the upper supports; see Figure 11.

Since the fiberoptic sensor was placed on the bottom of the CFW samples, the overall signal indicates positive strain values (elongation/tension), as expected. In contrast, the sensor on the top of the mōsō bamboo records negative strain values (contraction/compression). Two sections in the top signal display higher values that correspond to detour areas rather than actual measurements. These sections would be expected to appear more linear without the detour of the sensor, as shown by the dotted line (see Figure 11) if the sensor path had remained axial. The detours also artificially widen these regions in the visualization due to the increased sensor length in non-axial directions.

The average internodial strain in bamboo-inspired samples 1 and 2 is very similar, with minor deviations arising from fabrication tolerances characteristic of CFW. The maximum internodial strain in the CFW comparison sample is significantly lower, approximately half the level of the bamboo-inspired samples. The mōsō bamboo exhibits strain amplitudes exceeding those of the bamboo-inspired CFW samples. Furthermore, the strain distribution is asymmetric, with the left side showing higher values, likely due to uneven load introduction caused by geometrical irregularities or a reduced number of diaphragms per meter on that side of the mōsō bamboo sample. This asymmetry was not observed in the CFW samples, despite similar geometric deviations, indicating that the uneven strain distribution in bamboo is likely linked to irregular internodial lengths. The trapezoidal strain pattern expected in four-point bending is absent in sample 1 but can be observed in sample 2 and the comparison CFW sample, where internodial strain peaks are on the same level between the upper supports and decrease between supports on the same side. Strain measurements near the supports in the outer segments are unreliable due to boundary effects from the supports or lateral sensor placement.

In the CFW samples, the positions of the diaphragms are distinctly visible in the strain signal as local reductions to zero or even negative strain. In contrast, the mōsō bamboo displays a continuous, smoothed trapezoidal profile without local drops at diaphragm positions. The stronger effect by the segmentation in the CFW samples may result from differences in the relative ratios between wall and diaphragm thickness as well as internode-to-node diameter ratio. The comparison CFW sample also shows localized strain drops, suggesting that these reductions are associated with the general segmentation of the samples, which produces variations in diameter between internodes and nodes; see Table 5. While the fiber composite wall thickness remains constant, the structural depth increases at the nodes, reducing the strain values in the CFW samples. Comparing bamboo-inspired and comparison CFW samples shows that diaphragms attenuate the amplitude of node region deviations, thereby smoothing the signal; see Figure 11. In both bamboo-inspired and comparison CFW samples, a slope change is evident at the crossing point, reflecting variations in material cross-sectional distribution along the samples.

### 3.2. Failure Behavior

#### 3.2.1. Ovalization

Based on the anchoring concept (see Figure 5) the lateral surface of the CFW samples’ cylinder is subdivided into individual strands, which are connected only through the subsyntax 1.2 (see Table 3) via thinner strands. This configuration resulted in deformation of the internodes in the y,z-plane.

The nodes in the bamboo-inspired samples did not exhibit notable deformation prior to node failure, whereas the comparison CFW sample did, since it lacks diaphragms that could restrict deformations. Consequently, the comparison CFW sample displayed ovalization along the sample in only one direction, resulting in a flattening of the cross-section, including at the nodes, originating from the upper supports and extending along the entire length. This is supported by the literature [87], showing that higher fiber angles increase ovalization resistance.

**Figure 12:**
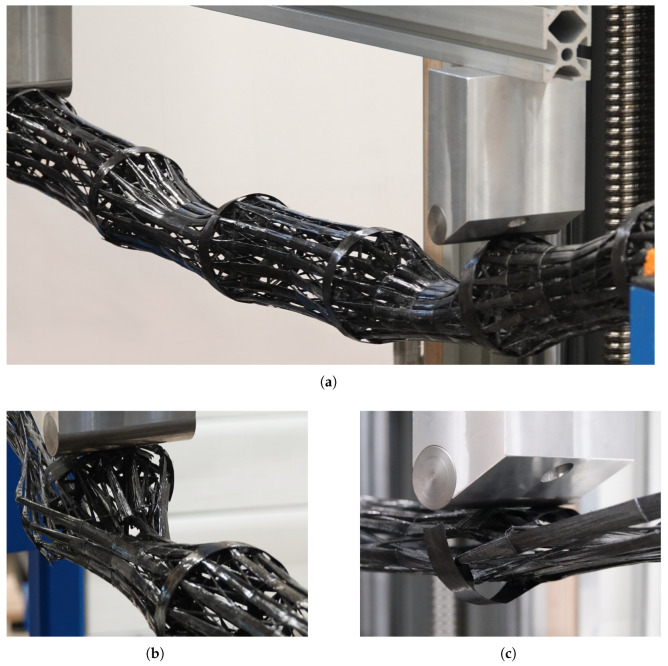
Photos of the structural failures. (**a**) Pattern of alternating internodial ovalization in the y,z-plane of the bamboo-inspired CFW sample. (**b**) Failure of the node in the bamboo-inspired CFW sample. (**c**) Failure of the node in the comparison CFW sample.

Bamboo-inspired CFW samples 1 and 2 demonstrated a pattern of alternating internodial ovalization in the y,z-plane, where the cross-section in the internode became elliptical, oriented horizontally in one internode and vertically in the next; see (a) in Figure 12. This alternating pattern arises from the mechanical coupling of the internodes enabled by the continuous fiber syntax. Manufacturing inaccuracies, in combination with the specific force introduction configuration, determined which internode deformed first and subsequently transferred this pattern to neighboring internodes. This explains why samples 1 and 2 exhibited alternating patterns that were inverted with respect to their orientation in the test setup.

Although this deformation contributed to the benign failure behavior and high energy absorption of the structures (see Section 3.3) suppressing such ovalization would significantly increase structural stiffness. Several strategies are possible: increasing the deposited material volume or enlarging the slits in the anchor disks until the individual strands merge into a continuous surface. Alternatively, additional material could be introduced at an angle, similar to subsyntax 1.2 or a newly defined equivalent. However, this would reduce the mass-specific stiffness due to the relative decrease of UD layers. The most effective option would be to wrap the entire structure with carbon fiber tape, resulting in a thin outer skin for shear transfer, combined with internal stiffening ribs, similar to [88]. All other samples did not exhibit notable ovalization across their lengths.

#### 3.2.2. Bending Shape

The steel square tube and mōsō bamboo displayed a bending line with uniform curvature along the entire length, as expected for four-point bending. The bamboo-inspired CFW samples approximated this bending line, albeit with deviations caused by manufacturing influences (compare Figure 11). The PVC tube and the comparison CFW sample did not show a uniform bending line: In the comparison CFW sample, later stages of structural testing revealed slight kinking near the upper supports due to substantial cross-sectional flattening at those node locations. In the PVC tube, the lower material hardness led the upper supports to indent the tube, causing a local cross-sectional reduction and thus producing a similar bending line with kinking at the supports, as expected [89].

#### 3.2.3. Structural Failure

The testing of the steel square tube and PVC tube was terminated before structural collapse occurred. In mōsō bamboo, continued testing beyond failure load caused partial structural damage, which was clearly audible, due to the fracture of individual diaphragms. At the ultimate load, two axial cracks developed on the upper side at approximately ±45° within the cross-section, originating from the left support (see Figure 11) and propagating inward, while the enclosed cylindrical surface bulged outward between the delaminations. A third axial crack emerged between them at the 0° position due to local buckling, extending the entire length between the upper supports. On the opposite side, a fourth axial crack formed along the underside at 180°, not exceeding the span of the upper supports. The internal diaphragms in this region were also fractured. Despite the severity of the damage, it remained locally confined compared to the CFW samples.

In the comparison CFW sample, the right node failed (see Figure 11) through fracture at ±90°, which subsequently facilitated cross-sectional flattening; see (c) in Figure 12. Alongside node failure and tape rupture, additional fiber breaks appeared at the top of the rightmost segments once the ultimate load was reached. This behavior indicates that diaphragms are essential for delaying ovalization and enabling benign failure. In the bamboo-inspired CFW samples, ultimate load was also accompanied by fiber fracture within the internodes, driven by critical local deformation; see (b) in Figure 12. Furthermore, delamination of the UD fiber layers from the diaphragm occurred at the load introduction zone, followed by fiber fracture in the bent fiber strands, and a rupture of the tape. Beyond connecting the individual UD strands, the failure behavior could be further improved through enhanced integration of the UD layers into the diaphragm layers.

### 3.3. Structural Testing Results

**Figure 13:**
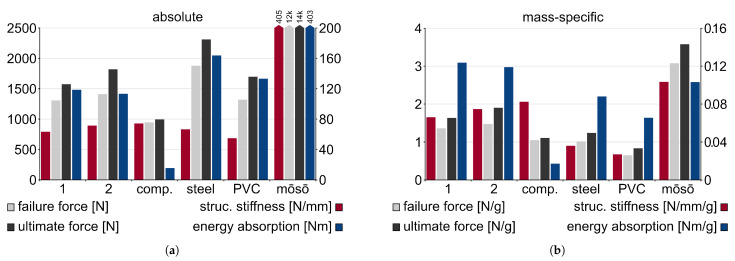
Structural testing results. (**a**) Absolute and (**b**) mass-specific results.

The load–displacement curves were analyzed to extract structural stiffness, failure load, ultimate load, and energy absorption; see Figure 13. The ultimate load was defined as the highest load recorded during the test, whereas the failure load was identified manually as the first structurally significant drop in load-bearing capacity without subsequent immediate stiffness recovery. To determine structural stiffness, each curve was divided into 40 equal bins between 2 and 22 mm displacement (24.6 and 28.6 mm for the mōsō bamboo). The slope of the load-displacement curve was calculated within each bin, and the maximum slope value across all bins was selected as the structural stiffness. The energy absorption was determined as the surface below the force–displacement curve between 0 and the ultimate load.

#### 3.3.1. Absolute Mechanical Performance

When comparing the absolute mechanical performance values, there is no significant difference in structural stiffness between the samples, with the bamboo-inspired CFW samples showing slightly lower values. However, for the failure and ultimate loads, the bamboo-inspired samples outperform the comparison sample by factors of approximately 1.4 and 1.7, respectively. This indicates that the introduction of diaphragms significantly increases the failure and ultimate load capacities that the structures can withstand without substantially reducing structural stiffness (90%). The positive effect of the diaphragms on the benignity is also reflected in the energy absorption capacity, which increases by a factor of 7.5 with the implementation of diaphragms in the CFW structure, while the comparison sample without diaphragms is insignificantly lighter due to their absence (0.95%). The CFW comparison sample exhibits the lowest energy absorption among all tested samples, reflecting the rather brittle failure behavior of the carbon fiber material when utilized as UD layers.

The steel square tube outperforms the bamboo-inspired CFW samples in both load capacity and energy absorption by roughly a factor of 1.4. In terms of structural stiffness, no notable difference was observed. However, the steel sample has approximately 1.9× the mass per meter compared to the bamboo-inspired samples.

The PVC tube shows a similar load capacity to the bamboo-inspired CFW samples and a slightly higher energy absorption (115%) but only about 80% of their structural stiffness, which is due to differences in material properties. The PVC tube is 2× heavier per meter than the average bamboo-inspired CFW sample.

The mōsō bamboo culm sample significantly outperforms all other tested samples in all metrics, reaching 6.0× the structural stiffness, 8.7× the failure load, 8.2× the ultimate load, and 3.5× the energy absorption of the bamboo-inspired CFW samples; however, the mōsō bamboo culm is also approximately 4.1× heavier per meter. Due to differences in sample geometry, clamping condition, and analysis methodology, the extracted structural performance indicators of the bamboo culm are not directly comparable to those found in the literature [90,91], but they are plausible.

#### 3.3.2. Mass-Specific Mechanical Performance

To compare mass-specific mechanical performance indicators, the mass per meter was used instead of total sample length, as the samples have different structurally relevant lengths; see Table 5.

The introduction of diaphragms in the CFW structure increases the mass-specific failure load by 36% and the ultimate load by 62% compared to the comparison sample without diaphragms. Thus, the advantage of the bamboo-inspired samples is reduced in the mass-specific comparison, as the comparison sample is lighter. Mass-specific structural stiffness of the bamboo-inspired samples is only about 86% of the comparison sample, indicating that diaphragm inclusion does not effectively increase stiffness in lightweight structures and mass would be more effectively utilized as additional UD layers. Nonetheless, energy absorption is still improved by a factor of 7, although slightly less pronounced than in absolute terms.

Regarding steel, the mass-specific performance is now inferior to the CFW samples due to steel’s much higher density. The mass-specific failure and ultimate loads of steel are both approximately 70% of those of the bamboo-inspired CFW samples. Steel reaches similar mass-specific load capacities as the CFW comparison sample without diaphragms, with a slight disadvantage in failure load but a small advantage in ultimate load. A substantial difference is, however, found in structural stiffness, where the bamboo-inspired samples outperform steel by a factor of 2, and the CFW comparison sample exceeds steel by a factor of 2.3, which demonstrates the superiority of the carbon fiber-based material system compared to steel. In terms of mass-specific energy absorption, steel also exhibits inferior performance relative to bamboo-inspired CFW samples at approximately 70%, highlighting the ability of the CFW fabrication process to create a fiber net with a more benign failure behavior. Here, the difference between material-driven samples (steel, PVC) and one with complex structural reinforcement is evident: the stiff unidirectional carbon fibers would reach ultimate load earlier if the diaphragms would not allow substantial reconfiguration, resulting in higher mass-specific energy absorption despite the brittle nature of the material itself.

The PVC tube underperforms in mass-specific performance indicators between 40 and 50% compared to the bamboo-inspired CFW samples, due to the absence of reinforcing materials competitive to steel or carbon fibers, combined with its relatively high mass. In comparison to the bamboo-inspired CFW samples, the PVC tube reaches only 56% in mass-specific energy absorption, again demonstrating the superior influence of the structural configuration over that of pure material parameters in defining the structure’s failure behavior.

Despite the high structural mass of the mōsō bamboo sample, it still substantially outperforms the other samples in mass-specific terms across all metrics except for the energy absorption (84%). This shows the superiority of the biological methodology optimizing in detail across all hierarchical levels. In mass-specific terms, the mōsō bamboo sample reached 1.5× the structural stiffness, 2.2× the failure load, and 2× the ultimate load of the bamboo-inspired CFW samples.

## 4. Conclusions

With the aim of enhancing mass-specific structural performance indicators, this study transferred structural design principles from bamboo culms into CFRP lightweight trusses fabricated by coreless filament winding. Thus, the CFW process has been enhanced by enabling the fabrication of structurally integrated diaphragms through a multi-stage tiling approach in combination with a water-soluble winding fixture. The developed multi-step numerical and analytical abstraction procedure was experimentally validated within a case study, comparing CFW samples in four-point bending alongside steel, PVC, and mōsō bamboo references.

In mass-specific terms, the integration of bamboo-inspired diaphragms increased the failure load by 36%, the ultimate load by 62%, the energy absorption by a factor of 7. However, this reduced the mass-specific stiffness by 14%, indicating that adding diaphragms improves benign failure behavior by influencing the axial strain distribution and thus increases failure capacity. To increase structural stiffness, the material should better be deployed in UD layers. When benchmarked against technical materials, the bamboo-inspired CFW samples outperformed steel and PVC samples in all mass-specific performance indicators. Nevertheless, the mōsō bamboo reference sample still surpassed all technical solutions, achieving 1.5× the structural stiffness, 2.2× the failure load, and 2× the ultimate load of the bamboo-inspired CFW samples, confirming the efficiency of nature’s hierarchical optimization.

The limitations of the presented approach are primarily linked to the increased fabrication complexity. The segmented approach would allow multiple workers faster fabrication during stage one. Furthermore, the notable differences between the targeted and actual geometrical parameters of the CFW sample provide potential for improvement and reveal that the trade-off between structural stiffness and load capacity, still needs adapting the design optimization weighting factor to specific applications. Thus, it can be expected that the mass-specific structural stiffness would increase with thicker walls and less/thinner diaphragms. Future work should focus on refining diaphragm-to-wall integration and improving shear transfer in internodes. Further optimization should be directed toward failure behavior rather than being limited to linear elastic analysis.

Beyond advancing composite fabrication techniques, this work highlights the value of bio-inspired and biomimetic design principles in engineering, demonstrating their potential for advancing lightweight structural design.

## Figures and Tables

**Table 1:** Structural features of the bamboo culm, their structural or otherwise biological function, and their technical adaptations in CFW fiber composite truss structures of the case study.

Features of the Bamboo Culm	Structural/Biological Function	Technical Adaptation in CFW
circular cross-section	direction-independent structural resistance	approximating a circular cross-section
hollow cross-section	allocating material where it is most efficient	tubular cross-section
homogeneous wall thickness	avoiding stress concentrations	approximating equal wall thickness
axial variation in outer diameter/ wall thicknesss.	keeping bending stresses constant	not integrated (sample too short)
structural segmentation by diaphragms	preventing ovalization of tubular structure	incorporation of diaphragms
internodal length variations	keeping bending stresses constant	not integrated (sample too short)
outer wall thickening at node	avoiding weak points	circumferentially reinforcing the node
shared fibers between diaphragm/internode	avoiding delamination between struc. members	uninterrupted fiber paths
several reinforcing schemes of VBs	resilient connection between struc. members	not integrated (too high complexity of process)
unidirectional fiber orientation in internode	maximize (bending) resistance	incorporation of unidirectional fiber layers
random fiber orientation in diaphragm	utilizing material’s tensile resistance	incorporation of fibers at different angles
VBs embedded in parenchyma cells	anisotropic material system	fiber-reinforced composite material
vessels bundled in VBs	nutrient and water transport	not integrated (not struc. significant)
sclerenchyma cells bundles in VBs	bundling for protecting VBs	not integrated (no intentional fiber bundling)
radial gradient of VBs in cross-section	allocating reinf. material where it is most efficient	approximated by the winding syntax
radial gradient in vascular bundle diameter	denser packing of VBs	not integrated (fibers with singular diameter)
epidermal layer	environmental protection of the structure	not integrated (env. prot. not relevant)
pith ring	transport of liquids	not integrated (not structurally significant)

**Table 2:** Parameters for the coefficients of the f(x)-approximation of $D_{t}\left(n,t\right)$; see Equation (2) and Figure 3.

Coeff.	For *a*	For *b*	For *c*	For *d*
*a*	3.126137 ± 0.07604	3.362831 ± 0.06932	9.314523 ± 0.0737	2.92553 ± 0.0808
*b*	0.792139 ± 0.2273	1.294047 ± 0.2058	3.321382 ± 0.6431	1.228597 ± 1.524
*c*	2.940157 ± 0.7278	16.25195 ± 7.037	431.5042 ± 9.938 $\times \;10^{3}$	428,455.3 ± 2.154 × 10^11^
*d*	2.725459 ± 0.03892	−1.329854 ± 1.544	1,221,961 ± 9.347 × 10^10^	−162,874.5 ± 1.006 × 10^11^
R^2^	100%	100%	99.97%	99.77%

**Table 4:** Fiber and matrix masses deployed during each stage of the fabrication.

Sample	Stage	Fiber	Matrix	FVR
1	1	153.2	127.2	42.8
	2	373.1	290.0	44.4
2	1	154.7	128.4	42.8
	2	374.8	275.3	45.8
comp.	1	159.5	90.3	52.3
	2	371.6	253.0	47.7
		g	g	%

**Table 5:** Mass and dimensions of the samples. The outer diameters were measured via the circumference. In the CFW samples, the total length excludes protruding fiber loops for fiber tension control. Internodial length between diaphragm wall surfaces; see Figure 1. In the CFW samples, the measured wall thickness neglects any additional structural depth caused by curvature of the fiber strands.

Sample	Mass	Length	Intern. Length	ØInternode	ØNode	Diaphragm Thickness	Wall Thickness
1	943.5	1736	216.03 ± 1.49	67.4 ± 2.7	91.5 ± 1.9	1.94 ± 0.17	3.77 ± 0.45
2	933.2	1755	218.27 ± 3.82	67.6 ± 4.7	92.2 ± 2.2	2.51 ± 0.33	4.26 ± 0.09
comp.	874.4	1717	429.25 ± 1.78	67.3 ± 1.0	93.6 ± 0.8	-	4.85 ± 0.30
steel	2100	2000	-	25.0 ± 0.1 (edge length)		-	1.50 ± 0.04
PVC	2300	2090	-	110.0 ± 0.2		-	3.20 ± 0.15
mōsō	4400	2000	198.27 ± 44.93	109.0 ± 5.6	110.9 ± 5.9	3.46 ± 0.23	11.80 ± 0.50
	g	mm	mm	mm	mm	mm	mm

## Data Availability

The data cannot be made publicly available upon publication because they are not available in a format that is sufficiently accessible or reusable by other researchers. The data that support the findings of this study are available upon reasonable request from the authors.
